# Bringing Value-Based Perspectives to Care: Including Patient and Family Members in Decision-Making Processes

**DOI:** 10.15171/ijhpm.2017.27

**Published:** 2017-03-06

**Authors:** Graeme Kohler, Tara Sampalli, Ashley Ryer, Judy Porter, Les Wood, Lisa Bedford, Irene Higgins-Bowser, Lynn Edwards, Erin Christian, Susan Dunn, Rick Gibson, Shannon Ryan Carson, Michael Vallis, Joanna Zed, Barna Tugwell, Colin Van Zoost, Carolyn Canfield, Eleanor Rivoire

**Affiliations:** ^1^Primary Health Care, Nova Scotia Health Authority, Halifax, NS, Canada.; ^2^Diabetes Management Centre, Primary Health Care, Nova Scotia Health Authority, Halifax, NS, Canada.; ^3^Public Engagement, Nova Scotia Health Authority, Halifax, NS, Canada.; ^4^Behaviour Change Institute, Primary Health Care, Nova Scotia Health Authority, Halifax, NS, Canada.; ^5^Dalhousie Family Medicine, Halifax, NS, Canada.; ^6^Endocrinology, Nova Scotia Health Authority, Halifax, NS, Canada.; ^7^General Internal Medicine, Nova Scotia Health Authority, Halifax, NS, Canada.; ^8^Canadian Foundation Healthcare Improvement, Ottawa, ON, Canada.

**Keywords:** Patient and Family Engagement, Decision-Making, Quality Teams, Patient Experience

## Abstract

**Background:** Recent evidence shows that patient engagement is an important strategy in achieving a high performing healthcare system. While there is considerable evidence of implementation initiatives in direct care context, there is limited investigation of implementation initiatives in decision-making context as it relates to program planning, service delivery and developing policies. Research has also shown a gap in consistent application of system-level strategies that can effectively translate organizational policies around patient and family engagement into practice.

**Methods:** The broad objective of this initiative was to develop a system-level implementation strategy to include patient and family advisors (PFAs) at decision-making points in primary healthcare (PHC) based on wellestablished evidence and literature. In this opportunity sponsored by the Canadian Foundation for Healthcare Improvement (CFHI) a co-design methodology, also well-established was applied in identifying and developing a suitable implementation strategy to engage PFAs as members of quality teams in PHC. Diabetes management centres (DMCs) was selected as the pilot site to develop the strategy. Key steps in the process included review of evidence, review of the current state in PHC through engagement of key stakeholders and a co-design approach.

**Results:** The project team included a diverse representation of members from the PHC system including patient advisors, DMC team members, system leads, providers, Public Engagement team members and CFHI improvement coaches. Key outcomes of this 18-month long initiative included development of a working definition of patient and family engagement, development of a Patient and Family Engagement Resource Guide and evaluation of the resource guide.

**Conclusion:** This novel initiative provided us an opportunity to develop a supportive system-wide implementation plan and a strategy to include PFAs in decision-making processes in PHC. The well-established co-design methodology further allowed us to include value-based (customer driven quality and experience of care) perspectives of several important stakeholders including patient advisors. The next step will be to implement the strategy within DMCs, spread the strategy PHC, both locally and provincially with a focus on sustainability.

## Background

Patient engagement is considered a key strategy to achieve the triple aim in healthcare, namely optimal experience, outcomes and efficiency.^1^ The definition of patient engagement can thus vary depending upon how patients are engaged in health system planning, such as in direct care, decision-making and system-level planning.^2-4^ Emerging evidence indicates that interventions that tailor support to the individual’s level of activation are effective in increasing patient engagement.^5^ Consequently, there appears to be a good number of initiatives and research work to understand the impact, implication and importance of engaging patients in direct care with the broader goal of improving health, outcomes and costs.

There is also growing awareness that patient and family engagement is a value-added and essential enabler to redesign and reform healthcare to better meet the needs of patients, families, and care givers.^6-9^ Consequently an emerging area of exploration in the patient and family engagement work is the inclusion of patient and family advisors (PFAs) in the decision-making context.^2-6^ Decision-making in this context refers to inclusion in decisions about programs/policies/service delivery versus decisions about personal care or choices. In some organizations, the central decision-making body around patient centered care (PCC) is a Patient and Family Advisory Council.^6^ A PFA works in partnership with a hospital/clinic/healthcare organization to create a truly patient- and family-centred care environment and experience. The PFA council is made up of senior clinical and administrative leadership, staff and PFA council members. These individuals come together to inform the direction and work of PCC and patient and family engagement within the organization. The ability to view service design and delivery through the patients’ eyes is important to ensure patient perspectives and priorities are maintained especially in the design of new programs.^3^ Although recognized as a highly supportive strategy in building a strong healthcare system, having PFAs in decision-making processes is not being consistently applied due to lack of consistent implementation strategies.^10^ These can include having a widely accepted definition that best represents and clarifies patient and family engagement in decision-making context, a process to recruit PFAs, interview guides to support recruitment, a process of orientation, supporting readiness strategies for teams to accept PFAs in the decision-making process to name a few.^3,6^

A patient engagement initiative in primary healthcare (PHC), Nova Scotia Health Authority (NSHA), addressed gaps and opportunities for creating a system-level implementation strategy to include PFAs at decision-making points in PHC.

### Problem Statement

PHC is a complex system with urban, sub-urban, and rural service locations, with team-based and individual practices, and a variety of payment plans and support services in the community. The complexity of the system prompted the leaders in PHC to review the patient engagement process across various service areas. Following an internal review, it was determined that developing a consistent value-based patient and family engagement strategy was essential to support a strong, effective and efficient PHC system. The opportunity to develop a system-level strategy to include PFAs in decision-making processes was realized in 2014 when PHC joined several teams across Canada Partnering with Patients and Families for Quality Improvement in a Canadian Foundation for Healthcare Improvement (CFHI) initiative. The improvement opportunity was to review and develop a locally contextual and meaningful patient engagement strategy in each partnering health authority.

### Objectives

The primary objective was to create a consistent system-level implementation strategy to include PFAs in decision-making points across programs and services in PHC through the inclusion of value-based perspectives.^7^ We define value-based perspectives in this work as customer driven values and perspectives on quality and experiences of care. Towards this broader objective, PHC set out to develop a systematic implementation strategy based on current evidence on patient engagement inclusion in decision-making context, current state of approaches in the health authority and inclusion of patient advisors in a co-design approach.

## Methods

### Key Phases of the Initiative

**Figure 1:**
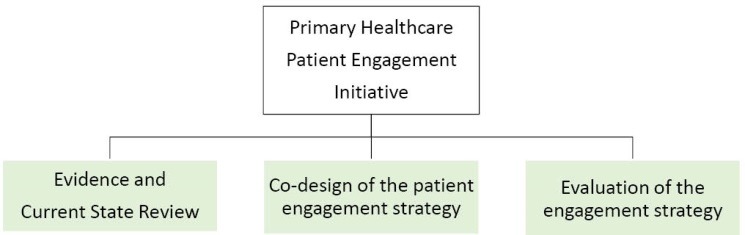
Phases of the Initiative

There were three main phases for the PHC initiative as shown in Figure 1. At the outset of the initiative, a comprehensive literature review and jurisdictional scan was completed to deepen our understanding of patient engagement. Jurisdicational scans are particular useful in policy- and decision-making process as they inform how a specific initiative or activity of interest has been framed, conducted or disseminated in other jurisdictions. They are particularly helpful in understanding implementation considerations, key learnings and potential pitfalls.

Through the literature review, jurisdictional scan, and a co-design methodology a strategy to create a consistent process for patient and family engagement across the PHC system was developed.

### Phase I – Evidence and Current State Review

Patient and family engagement has been called a critical part of a continuously learning health system, a necessary condition for the redesign of the healthcare system, the ‘holy grail’ of healthcare, and the next blockbuster drug of the century.^6^ As part of this initiative, we conducted a review of literature and a current status scan of patient engagement strategies in Canada within the health system.

“A literature search was performed using PubMed, Canadian Health Research Collection, CINAHL, ProQuest, NICE (Published Public Health Guidance and Standards and Indictors), Cochrane, Google and Google Scholar. Combinations of the following key search terms were used: patient and family engagement/involvement/participation, barriers and enablers to engagement/involvement/participation, shared decision-making, co-design, experience-based design, recruitment, selection, measurement and evaluation. Results from peer-reviewed articles and grey literature were included as well as a hand search of the reference lists and bibliographies of all retrieved articles. The Institute for Patient and Family Centered Care and Accreditation Canada’s Leading Practice Database was also reviewed. Supporting documents from healthcare organizations across Canada was used to support this work.” We retrieved and reviewed close to 30 articles of relevance to our search terms.

Based on the evidence review, we concluded the following. Despite its importance, there continues to be a lack of clarity of what patient engagement is, what it looks like, how it is measured and how it improves health outcomes for patients and families. In 2012, Gallivan et al^9^ conducted a scoping review which looked at the variation in terminology and the definitions used to describe patient engagement. As a result of the scoping review, 15 different terms related to patient engagement were found, including participation, involvement, consumer engagement, and public engagement. A lack of consensus and understanding about terminology, the goals and expectations and roles and responsibilities of stakeholders are major barriers to achieving meaningful and successful patient engagement. Patient and family engagement has also been characterized by the level of patient-provider communication, by the role of patients in their care decisions and by the involvement of patients in healthcare decisions and policy-making.^6^ Healthcare systems struggle with a tokenistic approach when engaging patients and families. Until healthcare systems are able to create a safe place where the voices of patients and families are valued, we will never properly engage patients and families. A shift is required within health systems to ensure patients and their family members can be more involved in decisions about their care.

Based on our evidence review, we identified the following key considerations while developing a system-level implementation to include PFAs at decision-making points: important considerations from evidence review of relevance to this initiative:

- Develop and establish a working definition that can support appropriate recruitment of PFAs relevant to the team/organization. Before considering patient and family engagement, there must be a clear understanding of what is meant by patient and family centered care and what type of patient and family engagement is relevant.^6-8^
- Create the right type of organizational level support and leadership is important to support teams, staff and PFAs that will be involved in the engagement strategy. The initiative must be engrained in the organizational strategy and must have high-level executive buy-in and support.^10^ It is important to spend adequate time in engaging formal leaders within the organization and establish support processes and strategies to improve sustainability.^4,6^
- Create a systematic strategy to support common goals and understanding of teams or staff in organization is important to ensure goals, outcomes and expectations are clearly outlined for all stakeholders including PFAs involved in the initiative or strategy.^9^
- Engage all relevant stakeholders including clinicians and staff in the engagement strategy. Clinicians and staff must have a good understanding/be supported in how to effectively engage with patients and families. Care team/committees and leaders need to know how to engage these advisors in an effective and meaningful way.^2,3^
- Include a strategy for active participation of PFAs involved in developing and implementing the strategy for patient and family engagement. There is clear evidence that most people want to play an active part in their own care and they expect health professionals to support them in this role.^8,11,12^ It is also clearly demonstrated that patients when included in direct care or decision-making strategies want to actively participate in the development and implementation of the strategy.^9,10^ This includes consideration of health literacy in the engagement strategy.^13,14^

A current state scan of local context and relevance to the health authority and PHC was also conducted. We primarily scanned patient and family engagement strategies within health systems in Canada (n = 9). The NSHA’s Act requires that the organization have a public engagement plan to engage and consult with the public in respect to the health services provided by the health authority. A Public Health Engagement Team has been created and since its establishment, strategic vision, mission and formal documents to support alignment at an organization level are in place. The Executive Leadership Team (ELT) within the organization as well as Senior Directors in various portfolios support the overall plan and strategy developed by the Public Health Engagement Team. Accreditation Canada also requires a focus on public engagement. As an accredited health authority, all leaders, staff, and physicians informally support this work. While the leadership support and global strategies for public and patient engagement were very strong within the organization, it was recognized that deliberate protocols for patient engagement at practice/team/program levels were not consistently present, applied or translated from the formal organization level strategy. The challenges related to this were similar to the ones identified in the literature in terms of translation and sustainability with a gap existing specifically in the area of including PFAs at decision-making points in PHC. This led to the subsequent efforts, methods, and strategy described in this paper.

### Phase II – Co-design of a Patient Engagement Strategy in Primary Healthcare – Piloting in Diabetes Management Centers

The literature review also helped identify methodologies that were considered best practice in developing and implementing patient engagement strategies. Patient co-design was identified as the most appropriate methodology to engage and include patient and family members’ perspectives in the design of a patient engagement strategy in PHC.^15-18^

**Figure 2:**
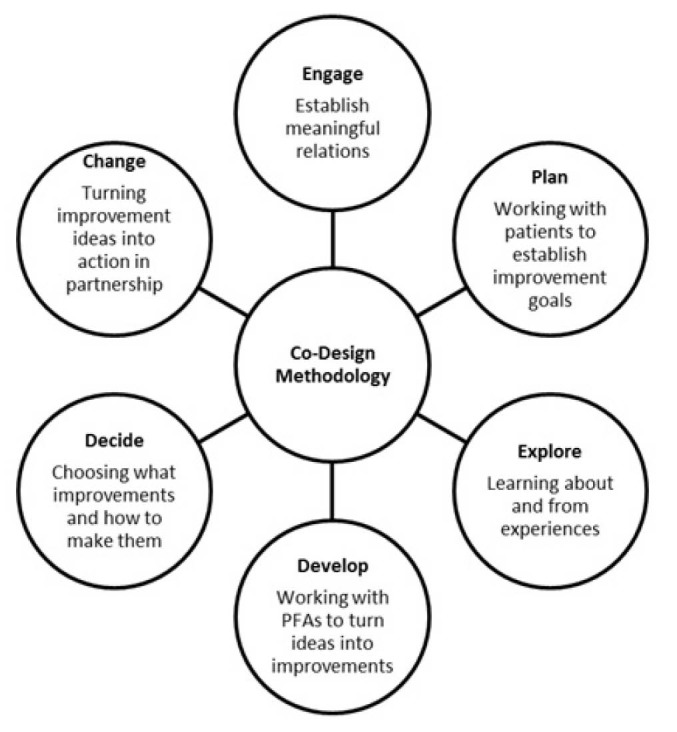
Co-design Methodology.^19^

In healthcare context, the term co-design refers to patients and families/carers working in partnership with healthcare staff to improve services.^16^ The work of the Waitemata District Health Board has clearly outlined key components of co-design methodology as shown in Figure 2 and described below.^19^

The diabetes management centres (DMCs) is a service area that falls within the PHC portfolio in central zone and spans rural and urban settings, it serves an increasingly complex patient population who are living with multiple chronic conditions. DMC team served as an exploratory site to support the development of the strategy in PHC with intent to spread across the PHC system in central zone.^20,21^

### Engage and Plan - Assembling a Team to Review and Co-design a Strategy

For this phase of work we were able to recruit primarily patient team members from the first few members that self selected to participate in the initiative. Family members were not approached to participate in this initiative. Three patients from the DMC service were ultimately recruited to participate in the co-design process. The full team also comprised of the formal leads of the initiative, PHC leadership members (Director, Medical Chief), DMC Manager, DMC Clinical Team Leader, a family physician, an internist, a behavioural psychologist, an endocrinologist, leads from the NSHA Public Engagement Team, and CFHI improvement coaches. Three levels of governance were created for members identified as relevant to the co-design process including (1) Core Design Team, (2) Implementation Team, and (3) Advisory Team. The core design team consisted of the project lead, project manager, evaluation lead, and 3 patient advisors. Following preliminary conversations, the core design team and the implementation team agreed to develop a guide to outline the general protocols and steps required to support consistent inclusion of PFAs in quality committees across the PHC system including a formal definition for the term “Patient and Family Engagement.” The team met monthly to co-design many of the tools and resources supporting the patient and family resource guide. The advisory team consisted of champions and leaders internal and external to PHC that could provide feedback as well as support the spread and sustainability of this work. These stakeholders were formally engaged quarterly throughout this initiative and as needed to meet initiative objectives.

### Explore - Including the Voice of the Community

One of the deliverables of this work was to complete a focus group with patients and families living with diabetes in order to understand their experience of care. This was an opportunity to include other patients and families not formally connected to this improvement project as well as an opportunity to understand the experience of individuals accessing the DMCs. In structuring the focus group, we wanted to ensure that the questions were clinically relevant, appropriate, informative, and actionable by the DMC. The questions for the focus group were co-developed by the staff in the DMC as well as the core design team supporting the CFHI work.

The session was co-facilitated by a Public Engagement Advisor and a patient living with diabetes. A staff member within PHC was present to record conversations and to theme the thoughts shared by the patient and family participants. A graphic facilitator was also present to visually record the conversation. Comments and feedback were modified slightly in order to ensure anonymity of the patient/family member and the staff of the DMC. Approximately 11 members participated in the focus group. Key themes brought forward by the community included (*i*) living with diabetes, (*ii*) education, (*iii*) care delivery, (*iv*) philosophy of care, and (*v*) involvement in decision-making. Table 1 shows summary of patient perspectives under emerging themes, feedback offered and how some of the feedback has been either incorporated or used as raising awareness information by DMC staff. A total of individuals with diabetes participated in the focus group.

**Table 1:** Summary of Focus Group Feedback

**Emerging Themes**	**Comments From Focus Group Participants**	**How It Was Actioned or Incorporated in DMC Process for Review and Consideration**
Living with diabetes	• Diabetes is an insidious disease. • A lot of information is provided when newly diagnosed. Need help with sharing this with family and friends so that they can adequately support. • Family and caregivers need to be integrated into care delivery. • A discussion around roles and responsibilities at the onset of diagnosis is important between provider and patient. • Self-management is critically important.	• Overall, this feedback will be brought up to the DMC quality team to explore options for improvement and review of current processes that address feedback. • Conversations with other healthcare leaders (managers, privacy officer) about inclusion of family members and engagement are ongoing. • Self-management • Always invited family and support person to sessions, this is written in letters. • Always get approval from patient and how much patients want to engage with families.
A different disease	Type 1 and type 2 are very different diseases • The experience of care is different. The challenges and stresses are different. • The approach that the healthcare team needs to take is different. • The information/education provided to patients is different and must reflect the population served (ie, gender and age of patient).	• Information is provided is different based on diagnosis – DMC team will review further to ensure clarity in information provided. • DMC team is currently engaged in emerging adult with type I diabetes study where key question such as what their experiences were, how they would like to engage in future. management of their own diabetes (phone, text, email, etc) are being explored.
Education	• Information needs to be updated and be current (pamphlets and materials). Information on current technologies and medications should be readily available. • Peer to peer learning and mentorship is valuable. • Education is needed across programs/services and portfolios in diabetes care (ie, ED etc).	• Planning upcoming changes to the website design. Plan to incorporate PFA in planning of how DMC information is shared on the website. • Always update and offer most recent pamphlets • One PFA is expressing strong interest in developing mentorship and peer to peer learning opportunities for patients. • Team lead is in communication with ED nurses, she provides targeted education, providing packages of resources, etc. It is outside the scope of DMC to educate new ED staff.
Philosophy of care	• “I am not solely a diabetic – I am a person living with diabetes. It does not define me.”	• Language in DMC is always “person with diabetes” not “diabetic.” • Plan to offer education / reinforcement messages. PCC to DMC team members at relevant team meetings will continue to be in place.
Care delivery	• Focus on professionalism and customer service. • Managing distress/stress, anxiety, emotions, exercise, well-being and other psychosocial challenges should be integrated into care delivery. • Communication between providers and staff within the team and across programs and services. • Consistency in the delivery of care across locations and sites. • Feedback and quality improvement initiatives from the patient perspective should be included and actioned.	• DMC team is currently participating in multiple research initiatives that plan to review and address diabetes distress screening and supports, behaviour change training sessions to staff, self-management tools and resources for patients. and staff New electronic documentation is hoped to address communication among providers. • Three patient advisors now sit on DMC quality team to identify and support ways to include patient perspectives in DMC care delivery processes.
Access	• Opportunities to connect with staff outside of clinic appointments is critical in supporting self-management. • Other methods of communication (ie, email and texting) would be welcomed.	• Opportunities to strengthen DMC strategy for self-management will be clearly articulated and communicated to patients. • Extend offer to support via phone call. • Due to privacy there are considerations that need to be accounted for when communicating with patients via texting and email.

Abbreviations: DMC, Diabetes management centre; ED, Emergency Department; PFA, patient and family advisor; PCC, patient centered care.

Emerging themes and comments are provided below. The DMC and PHC staff have reviewed feedback and have assigned steps/process to address feedback received as outlined in Table 1.

Listed below are examples of participant perspectives about being involved in decision-making processes:

- Felt that the management of my disease was very much a discussion and a conversation. It is a 2-way street and partnership with staff. Although these reflections are accurate for some patients, this experience was not shared by all patients.
- I feel like I can ask questions openly and get an answer – the healthcare team is responsive to my questions or concerns.

### Develop and Decide

Based on the initial conversations with the patient advisors, the full team and the community engagement, the following were identified as necessary steps in the creation of a PFA engagement strategy in DMC and PHC.

1. Developing a working definition
2. Development of resource guide to support implementation of PFA engagement strategy across DMCs and PHC using the co-design methodology
3. Evaluation of resource guide

The implementation and evaluation of the implementation are currently being evaluated and will constitute the change part of the methodology which will be discussed in subsequent publications.

## Results

### Outcome 1 Development of a Working Definition for PFA Engagement Strategy in PHC

Co-design of a working definition with the help of patient advisors: We define engagement for our healthcare community (patients, families, caregivers, providers, health system and community) as “the active involvement and development of meaningful partnerships that respect the mutual knowledge and expertise of all involved leading to better care experiences.”

### Outcome 2 Co-design of a Resource Guide for PFA Engagement in PHC

Six stages of co-design methodology were applied to identify a process to standards PFA engagement in PHC as shown in Table 2.

As described in Table 2, there are 6 phases in the co-design of the resource guide. As part of the first part of our initiative, we completed 5 phases of the co-design and the results have been presented in this paper. The sixth phase, namely, the Change phase is the implementation of the resource guide to include PFAs as equal members of quality teams within DMC and other service areas and programs in PHC. The results from the 60 phase will be presented in a subsequent article.

**Table 2:** PHC Co-design Elements

**Co-design Elements**	**Waitamata Design Principles**	**Application of Design Principle in PHC Initiative**
Engage	Establishing and maintaining meaningful relationships with patients to understand and improve healthcare services.	Recruitment of patient advisors and the design team
Plan	Working with patients and staff to establish the goals of the improvement work and how one might go about achieving them.	Establish key elements of project and strategy – project objectives, patient engagement reference definition, outcomes, process
Explore	Learning about and understanding patient experiences of services and identifying improvement ideas.	Engaged broader diabetes community in an experience-based design conversation to learn about experiences of services and opportunities to engage
Develop	Working with patients to turn ideas into improvements that will lead to better patient experiences.	A guiding document to support the inclusion of PFAs at decision-making tables : co-design of the Resource Guide
Decide	Choosing what improvements to make and how to make them.	Development of implementation and evaluation strategies
Change	Turning improvement ideas into action in partnership with other stakeholders.	Design team and PHC leadership team will implement resource guide in DMC and create a plan for uptake in other service areas

Abbreviations: DMC, Diabetes management centre; PHC, primary healthcare; PFA, patient and family advisor.

Key components of the resource guide were developed in consultation with the design team and through the focus group. The components and highlights of elements developed in each component are described in Table 3.

**Table 3:** Components and Elements of the Resource Guide

**Components of Resource Guide**	**Key Elements Developed in PHC Initiative Using a Co-design Approach**
Patient and family engagement definition	A definition for patient engagement that meets the local context and needs
Patient engagement strategy for teams	Orientation and overview document for teams, Engagement 101 presentation, patient community engagement protocols, Terms of Reference document, PFA intake flow document
Readiness assessment tools	Managers, teams, and staff
PFA recruitment steps	Position description, roles and responsibilities, interview questions, orientation checklist
Team engagement tools	Reflection tool (pre-assessment survey for teams), Engagement 101 presentation
Evaluation tools	Evaluation framework and access to relevant surveys and tools

Abbreviations: PFA, patient and family advisor; PHC, primary healthcare.

### Outcome 3 Evaluation of the Resource Guide

The resource guide was reviewed for overall quality, content, context, flow and applicability by several different members of the project team, PHC system and NSHA (n = 15). A high level overview of the comments that helped further improvement of the resource guide content is presented in Table 4.

**Table 4:** Evaluation of the Resource Guide

**Participants**	**Feedback Offered and Revisions to the Resource Guide**
PHC Leads, PHC project sponsor and advisory committee members	Navigation, flow, and language Feedback on keeping the resource guide easy to read, navigate and simple in content. Maintain a “how to guide” approach. Length of the guide was identified as a concern and potential barrier for implementation. Lack of consistency in language, evidence supports and general flow was identified as an issue and a barrier to the use of the guide. Revisions: The resource guide language was simplified to read more like a “how to document” versus a research document. Language in the guide was made consistent and flow was improved by reorganizing sections within the guide.
NSHA Public Engagement Advisor	Context and relation to other priorities and initiatives Feedback focused on including context, relevance and relation to the broader policies and protocols included in the Public Engagement initiative in NSHA, alignment of language and content with Guide to Effective Engagement developed by the Public Engagement Team of NSHA, Accreditation Canada standards and the Health Authority Act in Nova Scotia. Revisions: A preamble was provided in the guide to establish the overall context, relevance and alignment with relevant standards such as Accreditation Canada and organizational strategy for public engagement.
PFAs	Language, navigation Feedback focused on plain language/readability and length of the resource guide. Revisions: A comprehensive report outlining the evidence, methodology and results was created so these elements could be removed from the resource guide keeping it focussed on the steps involved in implementation. This has created a marked improvement in the flow, focus, simplicity and application of the guide.
CFHI coaches	Content, context, and relation to other priorities and initiatives Feedback focused on clarity of definition included in the guide along with the content (clarity in articulating evidence in the literature, NSHA context, alignment with Accreditation Canada and quality and safety initiatives). Feedback to strengthen the content outlining the benefits from an organization/health authority perspective. Revisions: A preamble was included to establish alignment with organizational strategy and priorities and Accreditation standards. An introductory section was included to define key terminology used in the guide such as PFAs, shared decision-making. A paragraph has been included to outline the benefits to the organization and staff.

Abbreviations: PFA, patient and family advisor; PHC, primary healthcare; NSHA, Nova Scotia Health Authority; CFHI, Canadian Foundation for Healthcare Improvement.

## Discussion

This collaborative provided us the opportunity to develop an infrastructure for the inclusion of patient and family members in decision-making processes to support the development and enhancement of a strong PHC system.^22,23,25-27^ Patient perspectives and involvement at this level of engagement is considered a triple aim strategy for the healthcare system.^24,25^ The next step will be to implement the strategy within DMCs, spread the strategy PHC, both locally and provincially with a focus on sustainability and inclusion of public participation in policy-making.^26,27^ This will include the “Change” part of our initiative (Figure 2).

This patient engagement initiative has developed a structure to establish a consistent strategy at a system level in PHC for including PFAs in decision-making processes. Specifically, our focus was to create a strategy to include PFAs in quality teams within PHC initiatives and services. Quality, safety, and risk management is identified as a core enabler of a strong PHC system in the literature.^19^ As such, PHC maintains a focus on supporting quality at the system level and at the program and service level through the establishment of quality and safety teams, composed of clinical staff, clerical, staff, and administrative leaders. At the team level, quality and safety teams support the review of program/service specific data, processes, client/patient feedback, staff and patient safety, and team-specific quality improvement initiatives guided by a plan-do-study-act approach, among numerous other activities. The quality and safety teams also work in conjunction with a PHC joint occupational health and safety committees. From a leadership and accountability perspective, the program and service level teams are accountable to an overarching Primary Healthcare Quality and Safety Council at the departmental level, which is in turn accountable to the portfolio Senior Director and Vice President. As a starting point for this strategy, a PFA resource guide has been co-designed. Subsequent steps will involve the implementation and spread the application of the guide to recruit PFAs as members of quality teams in services and initiatives across PHC system. Quality teams in PHC are an ideal platform for PFAs to engage in decision-making activities around program planning, policies and service delivery.

## Conclusion

This improvement initiative set out to create the necessary tools, resources and infrastructure to support the development of a strategy to ‘collaborate and empower’ with PFAs at decision-making points in PHC. The resource guide developed through this novel initiative will help guide the next steps of implementation and spread across the PHC system. The long-term goals of this initiative are to support patient-centered care delivery and enhancement of health outcomes.^26-28^

## Acknowledgements

We would like to acknowledge the amazing support and contributions from the CFHI team and improvement coaches, PHC and NSHA staff who offered their time and commitment to this wonderful work, and most importantly our citizens who offered valuable insights and feedback that helped shape this work.

## Ethical issues

This study was approved by the Nova Scotia Health Authority ethics committee.

## Competing interests

Authors declare that they have no competing interests.

## Authors’ contributions

GK, TS, and AR contributed towards the design, development, implementation and evaluation of the initiative and were the main contributors in the write-up of the manuscript. EC, LE, MV, JZ, BT, CC, ER, RG, and SRC contributed towards the design of the initiative, as expert consultants and coaches, and supported the write-up of the manuscript, and finally our patient advisors, JP and LW contributed towards the design, development and implementation of the initiative and the manuscript. CVZ contributed towards design and development and served as an expert consultant for the initiative.

## Authors’ affiliations

^1^Primary Health Care, Nova Scotia Health Authority, Halifax, NS, Canada. ^2^Diabetes Management Centre, Primary Health Care, Nova Scotia Health Authority, Halifax, NS, Canada. ^3^Public Engagement, Nova Scotia Health Authority, Halifax, NS, Canada. ^4^Behaviour Change Institute, Primary Health Care, Nova Scotia Health Authority, Halifax, NS, Canada. ^5^Dalhousie Family Medicine, Halifax, NS, Canada. ^6^Endocrinology, Nova Scotia Health Authority, Halifax, NS, Canada. ^7^General Internal Medicine, Nova Scotia Health Authority, Halifax, NS, Canada. ^8^Canadian Foundation Healthcare Improvement, Ottawa, ON, Canada.

## Key messages

**Implications for policy makers**

This paper described an important initiative that has applied a co-design approach to create a strategy to engage patient and family members at decision-making points. This strategy is considered a priority to achieve triple aim (patient experience, health outcomes and health system efficiency) and in creating a high performing system. The resource guide co-designed in this study has the potential to be applied across various settings including healthcare, research programs, and organizational development processes.

1. Important messages for decision- and policy-makers:
Patient and family engagement takes time and commitment.
2. Awareness and transparency of the type and level of engagement with patient and family advisors (PFAs) and relevant stakeholders is extremely important. A PFA works in partnership with a hospital/clinic/healthcare organization to create a truly patient- and family-centred care environment and experience.
3. Enabling relevant supports and strategies to engage PFAs are essential for spread and sustainability.
4. Strategic leadership and support is critical pillar for the above.
5. Early engagement of staff and team/program is important. Assessing readiness and providing supports to enhance readiness is equally important especially when including PFAs in decision-making conversations.

**Implications for public**

While the value of engaging patients in direct care context is being widely recognized, there are limited efforts in the inclusion of patient and family advisors (PFAs) in program planning, service delivery planning and policy development. There is growing awareness of the value that PFAs bring to decision-making processes based on their experience and value-based perspectives. We used a co-design approach working closely with patient advisors to help us understand, identify and develop a system-level implementation strategy in primary healthcare (PHC) with a broader vision to not only enhance but also to create a strategy for more public involvement in policy development.
